# Bridging one health and sustainable analysis: enrofloxacin quantification amid combined therapy and its active metabolite in various matrices using green RP-HPLC

**DOI:** 10.1186/s13065-024-01283-3

**Published:** 2024-09-19

**Authors:** Heba M. Mohamed, Mona T. Ragab

**Affiliations:** Analytical Chemistry Department, Faculty of Pharmacy, Cairo University, Kasr El-Aini Street, Cairo, 11562 Egypt

**Keywords:** Green analysis, Antibiotics analysis, Enrofloxacin, Pharmaceutical and biological samples, Greenness assessment

## Abstract

Antibiotics play a crucial role in the treatment of infectious diseases in both humans and animals. However, their extensive utilization has caused significant potential harm to both wildlife and humans. Enrofloxacin (ENR) is a common veterinary antibiotic, which is not approved for human use due to associated toxicities. It is often combined with other antibiotics to expand the antibacterial range. It is crucial to monitor and measure the levels of ENR medication in various matrices. RP-HPLC is highly effective for analyzing antibiotics due to its sensitivity, specificity, and ability to handle complex samples. By adopting eco-friendly solvents, decreasing solvent consumption, and limiting waste we developed a method for determination and quantification of ENR, amoxicillin (AMX), and ENR active metabolite in different matrices. The method utilized a reversed stationary phase and a mobile phase composed of phosphate buffer pH 3.0: ethanol (90:10 v/v) pumped at 1.0 mL/min and UV detection at 254.0 nm. Moreover, a comprehensive assessment of the environmental friendliness of the established method was conducted using various tools including the Green Certificate Classification (GCC) and Analytical Greenness AGREE and RGB12. The method was validated for its accuracy and precision in quantifying ENR, demonstrating its potential for the effective monitoring of ENR and contributing to public health protection.

**Graphical Abstract:**

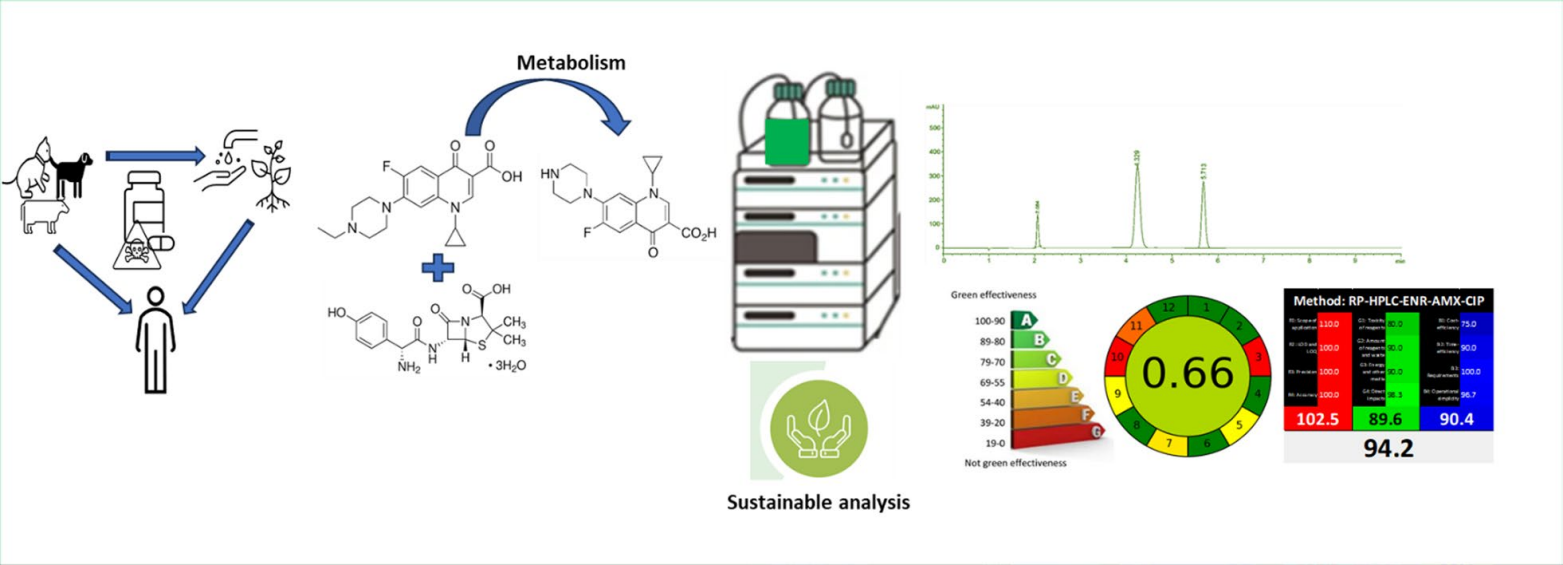

## Introduction

Antibiotics are essential for protecting against infections in humans and animals and are also used to improve animal growth and feed efficiency [1]. However, a significant amount of these medications, whether in their original form or as active byproducts, is released into the environment through various means [2]. Studies have shown that animal waste, such as feces and urine, can retain up to 90% of the antibiotics administered, mostly remain in their unchanged forms [3, 4]. This alarming contamination of the environment with antibiotics poses serious threats to both ecological balance and human health. Some of these antibiotics are even not approved for human use which intensifies their risk.

**Fig. 1:**
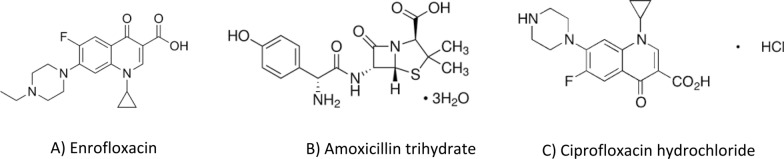
Chemical structures of **A** enrofloxacin, **B** amoxicillin trihydrate, and **C** ciprofloxacin hydrochloride

Fluoroquinolones (FQs) are a potent class of antibiotics used in both human and veterinary medicine but have recently faced regulatory restrictions. Enrofloxacin (ENR), a second-generation fluoroquinolone antibiotic (structure shown in Fig. 1), is highly effective in treating a variety of infections in animals [5, 6]. Its antimicrobial efficacy is enhanced by the formation of its active metabolite ciprofloxacin (CIP), (structure shown in Fig. 1) [7]. One of its key mechanisms of action is the inhibition of DNA gyrase enzymes, also known as topoisomerase II, which are essential for maintaining the structure of bacterial DNA [8]. Due to its excellent bioavailability, ENR is quickly absorbed through various routes in the body and primarily eliminated through renal excretion [9]. While it is a widely prescribed antibiotic for animals, ENR has not been approved for human use due to its potential to cause harmful side effects, such as gastrointestinal disturbance, other serious side effects like tendinitis, tendon rupture, central nervous system disturbances, and teratogenic effects [10–13]. A common veterinary combination for ENR is pairing and working in tandem with Amoxicillin (AMX), a beta-lactam antibiotic (structure shown in Fig. 1), where the therapeutic scope is greatly expanded. This synergistic combination ensures comprehensive coverage against gram-positive as well as gram-negative bacteria in the digestive, urinary, respiratory, and skin infections in cattle, cats and dogs [14, 15].

When present in soil, ENR undergoes a gradual degradation that may result in its prolonged existence [16–18]. Research by Abero et al. [19] revealed that this presence can span 3 months or longer. Such persistence of ENR in the environment has detrimental effects, such as harming soil bacterial communities, impeding nutrient fixation in plants, and hindering organic matter decomposition in soil [20, 21]. Moreover, there is cause for concern about human health risks due to the potential for ENR to reach humans indirectly through environmental contamination and accidental contact with animals, including common household pets like cats and dogs. The pressing nature of these concerns emphasizes the importance to develop an efficient and precise analytical tool that can accurately detect ENR in presence of other commonly combined drugs and in presence of its active metabolite in various context.

In scholarly literature, many studies focused on the individual determination of ENR and AMX separately [22–29] and few studies determine ENR in the presence of its active metabolite ciprofloxacin [30–33]. Recently, ENR was detected in edible eggs in Korea using LC–MS/MS analysis, the study revealed cross-contamination [34]. Electrophoretic-based procedures have also been reported [35, 36]. Only one study determined ENR in its binary mixture with AMX, yet without the active metabolite [37]. As far as our knowledge, there is no concurrent green quantification procedure for the monitoring of ENR and AMX in the presence of ciprofloxacin (main active metabolite of ENR). Traditional reported methodologies, such as chromatographic techniques (e.g., HPLC and LC–MS/MS), have demonstrated high sensitivity and accuracy [38]. However, these methods often involve complex sample preparation, lengthy analysis times, and the use of toxic solvents, raising concerns about their environmental impact, none of the reported chromatographic methods were keen to assess the greenness of their procedure [39, 40]. UV spectroscopic methods, while less complex, generally suffer from lower sensitivity and specificity, particularly when dealing with complex mixtures like ENR, AMX, and CIP with a sever spectral overlap, and in various matrices where interference from UV-absorbing material can affect the method selectivity [41]. Moreover, electrochemical methods, while effective for detecting individual compounds, and the wide range of application [42], sometimes struggle with selectivity, especially when compared to the chromatographic methods, in simultaneous multi-analyte detection. Especially in the presence of structurally similar compounds like ENR and CIP, significant design modifications are often required to enhance selectivity [43].

The objectives of our study are: first, to establish a green, timesaving, sensitive and accurate RP-HPLC methodology that allow rapid screening and precise determination of ENR simultaneously in the presence of AMX and its active metabolite (CIP) in bulk powder, pharmaceutical dosage forms and urine samples. Secondly, to evaluate the greenness of the developed procedure, particularly given the recent significant increase in solvent and reagent use and waste production in the pharmaceutical sector. The eco-friendliness of the developed method was assessed based on the 12 principles of Green Analytical Chemistry [39, 40]. Green Certificate Classification (GCC) [44] and Analytical Greenness AGREE algorism [45], have been utilized to estimate the greenness of our established method. Moreover, we assessed the whiteness of the suggested method [46], it incorporates three key aspects—the method’s validity (represented by red), its environmental impact (green), and its economic implications (blue). The whiteness level of our developed method has been evaluated using the WAC tool RGB12 [46].

## Methodology

### Instrumentation

The separation was carried out on an Agilant 1200 Infinity Series liquid chromatography, which is equipped with a quaternary gradient pumping system and UV detector, with autosampler injector and quaternary pump (USA). A Dr. Maisch C_18_ reversed phase column with dimensions (75 mm, 4.6 mm, i.d 5 µm) was used for the separation. Samples underwent degassing in a sonicator and were then filtered through membrane filters with a pore size of 0.45 µm, as well as syringe filters with a diameter of 0.22 µm (USA). Data analysis was performed using OpenLab ChemStation software (USA).

### Reagents and materials

An authentic sample of ENR cat no. j60023.06 (ENR) was Purchased from CORNAL LAB, Cairo—Egypt. Amoxicillin trihydrate and ciprofloxacin hydrochloride were kindly gifted from EIPICO, 10th Ramadan City—Egypt. Their purities were checked and found to be 100.25 ± 1.286 for ENR according to the reported method [33], and 100.26 ± 1.19, for AMX according to the reported method [37]. HPLC grade Ethanol (Sigma-Aldrich, Germany) and water, (New Human Power I, Korea). Market dosage form: Baytril 100 mg/mL® injectable 100 mg ENR/mL manufactured by Bayer HealthCare LLC, Kansas, USA.

### Preparation of standard and working solutions and synthetic mixtures

Stock solutions for ENR, AMX, and CIP were formulated by dissolving 50.00 mg of each drug powder in ethanol into a 50-mL volumetric flask (1.0 mg/mL). Serial dilutions of each drug stock solution were done using the mobile phase to prepare a range of working standard solutions. Various lab mixtures were formulated using different proportions of ENR and AMX to cover the commonly used therapy ratios along with various ratios of CIP (the main active metabolite for ENR). Exact aliquots of ENR standard solution (0.2–0.8 mL), AMX standard solution (0.2–0.8 mL), and CIP standard solution (0.1–0.4 mL) were dispensed into 20-mL volumetric flasks, and their volumes were completed to the mark using the mobile phase.

### Procedure

#### Chromatographic parameters

The chromatographic separation takes place using Dr. Maisch C18 column with the following dimensions (75 mm, 4.6 mm, i.d 5 µm). The analytes were separated using a mobile phase that is composed of (90:10 v/v) Phosphate buffer pH 3.0: ethanol (1 mL/min flow rate). The UV detector, set at 254 nm, was used for detecting the injected solutions, while maintaining the column temperature at the standard room temperature, 25 °C.

#### Development of calibration curves

Aliquots of ENR, AMX, and CIP stock solutions each with a concentration of 1.0 mg/mL, were dispensed into a series of 20 mL volumetric flasks and diluted with the mobile phase to achieve concentrations ranging from 5 to 80 µg/mL for ENR, 5 to 100 µg/mL for AMX, and 5 to 100 µg/mL for CIP These solutions were then subjected to the previously mentioned chromatographic parameters for analysis. For each concentration of ENR, AMX, and CIP, the mean peak area ratios (referencing external standards of ENR 80 μg/mL, AMX 100 μg/mL, and CIP 100 μg/mL) were graphed against their respective concentrations to construct the calibration curves.

#### Synthetic mixtures analysis procedure

The synthetic mixtures were analyzed using the same procedure outlined in step (2.4.2). The concentrations of each of the investigated analytes were determined using their respective regression equations using the peak area ratios of individual components in each lab mixture.

#### Pharmaceutical dosage form and urine analysis procedure

0.5 mL of the injectable solution (equivalent to 50 mg ENR) was accurately transferred into a 50-mL volumetric flask. The flask was then filled to the mark with the mobile phase and mixed thoroughly. From this solution, a precise quantity corresponding to 0.4 mg ENR was transferred into a 20-mL volumetric flask which was subsequently filled to the mark with the mobile phase and mixed well. Afterwards, the solution was injected into the instrument and the developed procedure was followed.

For the urine samples, accurately weight 0.045 g ENR into 100-mL volumetric flask which was then filled to the mark with the mobile phase and mixed thoroughly. Accurate volumes were then transferred from the prepared ENR solution into 10-mL volumetric flasks. Aliquots of AMX stock solution (1.0 mg/mL) and CIP stock solution (1.0 mg/mL) were added to the flasks, followed by the addition of 1 mL of dog urine and then diluted with the mobile phase to the mark to prepare two ENR concentrations equivalent to 5 and 45 µg/mL.

Additionally, the accuracy of the proposed method in various matrices was verified by applying the standard addition technique that involved spiking the prepared samples with small increments of ENR.

#### Greenness and whiteness assessment

For our developed methodology we adopted two metrics to assess the Greenness, one numerical based scale and other graphical based model. Moreover, to assess the alignment of the suggested method with the White Analytical Chemistry (WAC), we adopted RGB12 tool.

##### Greenness evaluation using the green certificate classification metric

The Green Certificate Classification (GCC) is an improved iteration of the Eco-Scale [44], which evaluates analytical techniques through a holistic approach for their environmental impact. The assessment considers factors such as the use of hazardous substances, energy consumption, waste and emissions. Each element is measured using a point-based system (penalty points), resulting in a color-coded letter ranking. Standing at the lead is the ‘A’ category, denoted by a dark green shade, symbolizing methods with less than 10 penalty points and titled as the most eco-friendly. On the other end of the spectrum, the ‘G’ category is highlighted in red, indicating an accumulation of over 81 penalty points and labeling methods as harmful to the environment [44].

##### Greenness evaluation using the analysis of greenness algorithm

The 12 Green Analytical Chemistry (GAC) principles are quantified on a scale from 0 to 1, and their average is depicted at the center of the AGREE pictogram. In addition, the importance of specific criteria can be highlighted by assigning them a higher weight based on their significance level. The AGREE tool displays its findings in a straightforward, clock-like pictogram, using a deep red color to symbolize a score of zero. The central part of this circular pictogram reflects the overall performance of the analytical method, with a dark green shade representing methods that are almost fully “green” and very close to a perfect score of one. The AGREE tool has made evaluating analytical methods easy with its consideration of key factors such as energy usage, automation, chemical safety, sample size and processing, waste production and management, measurement location, and analyst safety. What sets AGREE apart is its comprehensive inclusion of all GAC regulations. In addition, it is a user-friendly automated tool with free and simple AGREE software [45].

##### Whiteness evaluation using RGB12 metric

The principles of WAC ensure the precision of the analytical procedure, covering of all the 12 aspects of (GAC) and the economic efficiency of the method. By incorporating the influences of three distinct groups—the red group for efficiency, the green group for adhering to green chemistry principles, and the blue group for economic efficacy. Through the fusion of these primary colors, the method achieves a final score that represents its analytical purity [46].

## Findings and discussion

### Development and optimization of the proposed method

In our work, we employed the One Variable at Time (OVAT) approach to calibrate the chromatographic variables.

#### Column selection

**Fig. 2:**
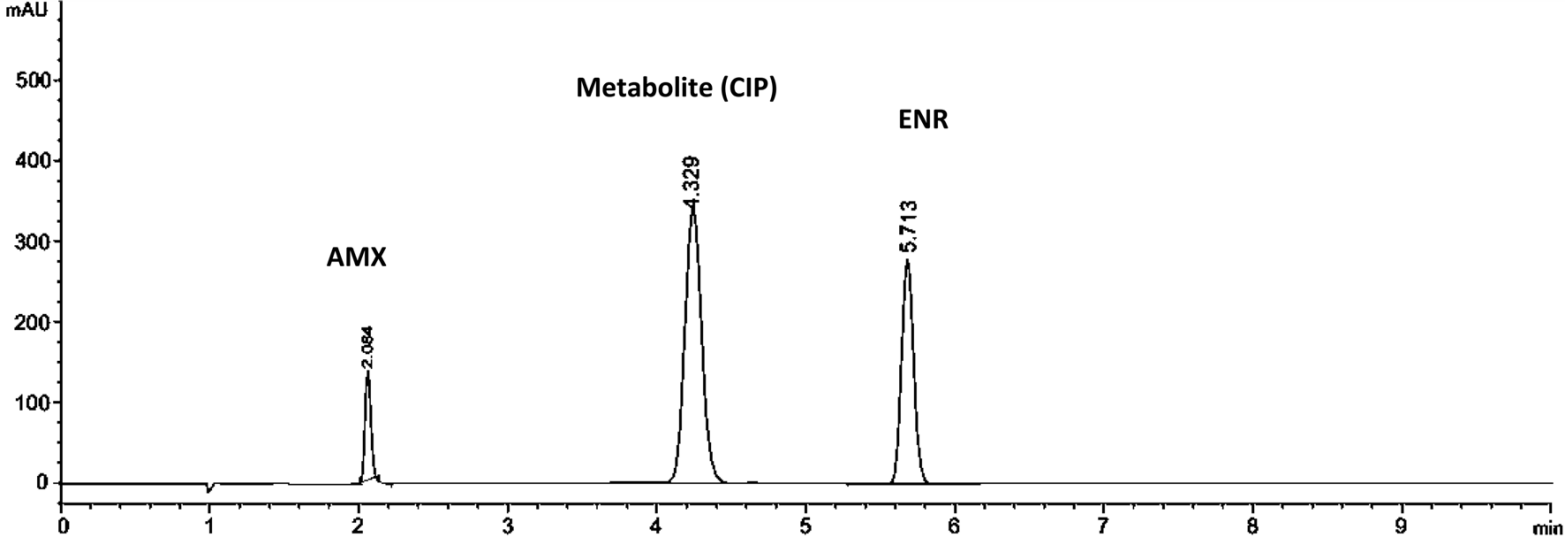
Chromatograms obtained for the separation ENR, AMX and CIP combinations using Phosphate buffer (pH3.0): ethanol (90:10 v/v) as mobile phase at 1 mL/min flow rate and detection at 254 nm

Achieving an optimal resolution (Rs) is a key objective in chromatographic analysis. Accordingly different reversed phase columns (C8 and C18) varying in length, particle size, and internal diameter were tried to validate the ability of achieving best resolution of ENR, AMX and the major metabolite (CIP). It was a bit challenging as ENR and CIP structures are highly similar, only differ in an ethyl group (Fig. 1). The C8 column failed in separating the three analytes properly, where the AMX eluted at a very short time interfering with the solvent peak while CIP and ENR came out as a split peak. Even with modification of the mobile phase no proper resolution was achieved. Other C18 columns were tried and succeeded in separating the three analytes, yet the peaks were tailed or with poor resolution or required lengthy separation time. Better resolution for these C18 columns was improved after increasing the organic modifiers (which conflicted with the main goal of the study of having greener separation option). The use of Dr. Maisch C18 column with the followings dimensions (75 mm, 4.6 mm, i.d 5 µm) as a stationary phase has enhanced the resolution compared to other tested reversed phase columns; using minimum percentage of ethanol (will be discussed in “Composition of the mobile phase” section). The peaks were nicely separated in less than 6 min with optimum resolution and selectivity as seen in Fig. 2. All experiments were appropriately conducted at ambient room temperature, 25 °C.

#### Composition of the mobile phase

**Fig. 3:**
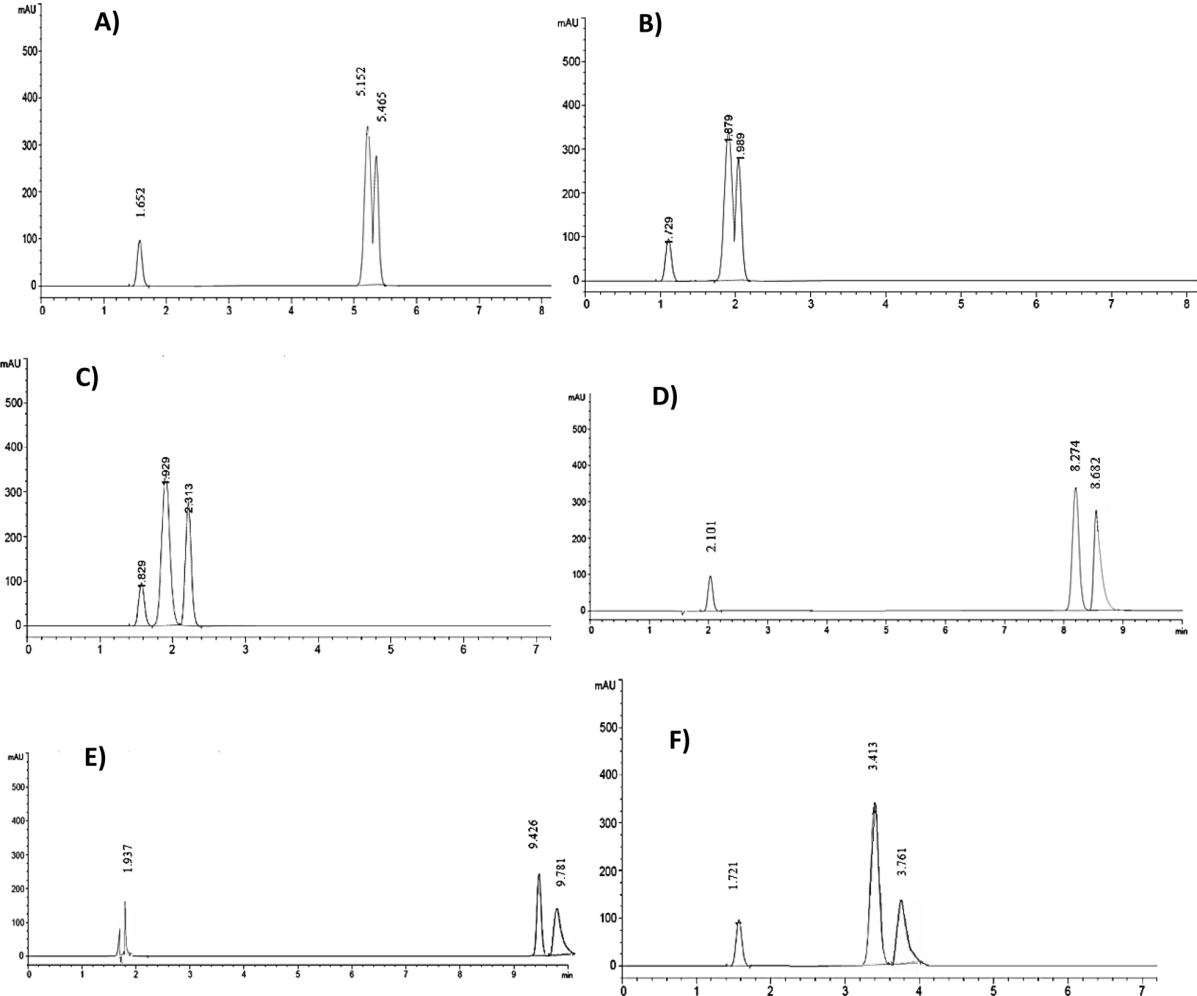
Chromatograms showed bad separation and retention of AMX, metabolite (CIP) and ENR. **A** Trial using RP-C8 and higher ethanol percentages. **B** Trial using RP-C18 and less ethanol percentages < 10%. **C** Trial using RP-C8 and less ethanol percentages < 10%. **D** Trial using RP-C18 and mobile phase including heptansulphonate and buffer pH with ethanol. **E** Trial using RP-C18 and mobile phase buffer pH 7.0. **F** Trial using RP-C18 ethanol using phosphate buffer pH 2.8

Ethanol was chosen as a less hazardous organic modifier to reduce the harmful effects of other solvents such as acetonitrile and methanol [39], and it successfully enhanced the separation of the analytes within an acceptable run time. Various ratios of phosphate buffer pH 3.0 and ethanol were experimented in both isocratic and gradient elution modes. Isocratic elution effectively separated the two analytes along with the main metabolite. The primary trials tested higher percentages of ethanol, gradually increasing up to 40%. However, as the ethanol percentage increased, the separation between ENR and CIP worsened and resulted in raising the column pressure (Fig. 3A). On the other hand, decreasing the ethanol percentage to less than 10% led to early elution of the three analytes within 1–2 min with overlapping peaks due to polarity of the analytes (Fig. 3B, C). Including an ion pairing agent, such as heptansulphonate, within the mobile phase was also tried, yet the resolution was not successful and it caused a delayed elution, especially for the CIP and ENR (Fig. 3D).

The selected composition for the isocratic solution consisted of 90:10 ratio of phosphate buffer (pH.3): ethanol gives a very optimum peaks resolution in a considerably short time (Fig. 2). The gradient elution method added complexity without improving separation, hence, isocratic elusion was preferred.

Furthermore, we explored the effect of altering pH levels of the phosphate buffer (ranging from pH 2.8 to pH 7.0) on the retention times and resolution of the three analytes. It revealed that a pH of 3.0 provided the best retention times and resolution. ENR and its active metabolite (CIP) are amphoteric compounds that exist in their ionic forms at both acidic and basic pH levels relative to their pKa values. It was observed that ENR and CIP had more retention at pH levels higher than 5, with tailing peak and deteriorated separation quality. The impact of pH on the retention time of AMX was minimal (Fig. 3E, F). The flow rates were also tested 1.2, 1.0, and 0.8 mL/min were tried, and1.0 mL/min identified as the most efficient, balancing pump pressure and peak separation. Accordingly, our study achieved significant outcomes in developing an eco-friendly analytical method by optimizing the mobile phase composition to ensure effective separation of the analytes while minimizing the use of hazardous solvents.

#### Selection of wavelength for analytes detection

Choosing the right wavelength is vital since it impacts the sensitivity of the method. The spectral profiles of the drugs under investigation were reviewed and the reported methods were compared in order to help finding the most suitable wavelength for the detection of the three components. For the ENR, it has its maximum absorbance at 278 nm and other λ_max_ at 316 and 330 nm, with the highest absorption at 278 nm, and for AMX, its λ_max_ is 230 nm [47, 48]. While for CIP its λ_max_ is 277 nm [49]. Upon trying the measurement at 276 nm, the AMX peak was not detectable unless its concentration was significantly high, which negatively impacted peak resolution and symmetry, as well as reduced the method’s sensitivity. When measuring at 230 nm, ENR and CIP were not clearly detected and optimal peak separation was not achieved. Accordingly, the UV detection at 254 nm was determined to be ideal for both visualization and quantification of all the three components, as it provides a high signal-to-noise ratio and a large peak area for the three analytes [50]. This contributes to the simplicity and practicality of our method, making it adaptable to various laboratories.

### Method validation

The developed method was validated in accordance with the guidelines of the International Conference on Harmonization (ICH) [51] linearity, accuracy, specificity, repeatability and intermediate precession were checked as shown in (Table 1).

**Table 1:** Linear regression and validation parameters obtained by the proposed RP-HPLC

Linearity	ENR	AMX	Metabolite (CIP)
Range (µg/mL)	5.0–80.0	5.0–100.0	5.0–100.0
Slope	0.0128	0.0101	0.0102
Intercept	− 0.0221	− 0.0123	− 0.0207
Correlation coefficient (r)	0.9997	0.999	0.9998
Accuracy^a^ (mean ± SD)	100.12 ± 1.041	100.59 ± 1.138	99.66 ± 1.376
Precision^a^ (RSD%)			
Repeatability	100.65 ± 1.411	99.38 ± 1.079	100.28 ± 1.406
Intermediate precision	100.55 ± 1.238	99.57 ± 1.549	100.12 ± 1.383
LOD^b^ (µg/mL)	1.072	1.372	1.345
LOQ^b^ (µg/mL)	3.25	4.11	4.11

^a^Average of nine determinations of three concentration levels

^b^Limit of detection and limit of quantitation were determined using signal/noise procedure (S/N = 2,3 for LOD and S/N = 10 for LOQ)

The calibration of the assay was done within the ranges of 5.00–80.00 μg/mL for ENR, 5.00–100.00 μg/mL for AMX, and 5.00–100.00 μg/mL for CIP.

The equations representing the linearity curves are as follows: A_ENR_ = 0.0128 C − 0.0221, r = 0.9997, A_AMX_ = 0.0101C − 0.0123, r = 0.9999, A_CIP_ = 0.0102C − 0.0207, r = 0.9999, where A is the peak area ratio of the analyte, C is the concentration of the analyte in μg/mL, and r stands for the correlation coefficient.

Applying the suggested methodology to analyze the pure forms, synthetic prepared mixture, pharmaceutical formulations, and urine samples, revealed non-significant interference as shown in (Table 2) and illustrated in (Fig. 2). The calculated LOD and LOQ (Table 1) of the developed method are low enough to detect traces of ENR in urine after proper dilutions (calculated on 5 mg/kg dose for dogs and cats) as ENR mainly excreted in urine unchanged (60%) [52].

**Table 2:** Determination of ENR, AMX and CIP in laboratory-prepared mixtures and determination of ENR in marketable sample, dog urine and application of standard addition technique

Sample	ENR (mean ± RSD%)	AMX (mean ± RSD%)	CIP (metabolite) (mean ± RSD%)
Synthetic mixtures (n = 5)^a^	100.67 ± 1.572	99.75 ± 1.739	100.51 ± 1.470
Pharmaceutical DF (Baytril 100 mg/mL®)^b^	101.37 ± 1.486		
Standard addition of dosage form^c^	100.91 ± 0.814		
Urine sample			
ENR 5 µg/mL and AMX 5 µg/mL^b^	99.86 ± 1.876	No interference	No interference
ENR 45 µg/mL and AMX 50 µg/mL^b^	98.85 ± 1.146	No interference	No interference
Standard addition of urine sample^c^	100.35 ± 1.589		

^a^Average of three determinations

^b^Average of three determinations

^c^Average of determinations after three levels standard addition, using (5.0, 10.0 and 20 µg/mL ENR)

System suitability tests which included assessments of retention time (Rt), analytes resolution (Rs), selectivity, column efficiency and tailing factor were also conducted. The calculated parameters are detailed in (Table 3).

**Table 3:** System suitability parameters calculated for the separated three components by the proposed HPLC method

Parameter	AMX	CIP	ENR
Retention factor (k′)	1	3.3	4.7
Selectivity factor (α)	3.3		1.42
Resolution (R_s_)	9.2		4.6
Peak symmetry	0.92	1.12	1.0
Number of theoretical plates (N)	2844	2527	8317

Robustness was checked to confirm the resilience of the proposed method against minor intentional variations in experimental conditions, it was evaluated against various separation conditions such as varying organic modifier percentage, buffer pH, flow rate and wavelength. The developed method consistently produced satisfactory resolution. These conclusive results, detailed in (Table 4), showcase the method’s high robustness.

**Table 4:** Robustness of the proposed HPLC method

Parameter/analyte	AMX	CIP	ENR	AMX	CIP	ENR
	Ethanol % + 1			Ethanol% − 1		
T	0.94	1.20	1.12	0.92	1.10	1.10
K’	1.20	3.40	4.90	0.95	3.00	4.50
Rs		9.00	4.50		9.40	4.80
^a^Recovery %	101.34	99.98	100.95	100.62	99.62	101.28

	pH 2.8			pH 3.2		
T	0.92	1.14	1.14	0.90	1.20	1.00
K’	1.10	3.50	4.70	1.00	3.40	4.50
Rs		9.20	4.60		9.00	4.80
^a^Recovery %	99.82	101.05	98.83	100.14	100.01	99.74

	Flow rate (1 + 0.2 mL/min)			Flow rate (1–0.2 mL/min)		
T	0.94	1.10	0.99	0.90	1.20	1.10
K’	0.95	3.10	4.50	1.30	3.50	5.00
Rs		9.40	4.90		9.20	4.80
^a^Recovery %	99.83	100.46	98.92	99.36	100.68	99.21

	Wavelength 254 + 2 nm			Wavelength 254 − 2 nm		
T	0.92	1.12	1.00	0.92	1.10	0.99
K’	1.00	3.30	4.70	1.00	3.30	4.70
Rs		9.20	4.60		9.20	4.60
^a^Recovery %	101.56	99.39	98.63	100.74	100.7	100.3

^a^% assay was calculated from the regression equation

### Application on pharmaceutical and urine samples

The method developed was effectively applied for the assay of ENR in its pharmaceutical preparation Baytril 100 mg/mL® and spiked urine samples. Recovery percentages were found to be optimum, confirming the appropriateness of the proposed method for regular quality control testing of ENR and for ENR monitoring in biological samples in the presence of AMX in their combination therapy along with its active metabolite (CIP).

ENR is predominantly eliminated through the kidneys, with a significant amount of the original drug and its byproducts found in the urine [52–54]. In dogs given a dose of 5 mg/kg, the serum half-life of ENR is 2.73 h, and around 60% of the administered dose is excreted as unaltered ENR, with the rest being excreted as metabolites, including ciprofloxacin [45, 46]. These urine values demonstrate the suitability of the developed method to detect traces of ENR in urine after proper dilutions, confirmed by the methods LOD (1.072 µg/mL) and LOQ (3.25 µg/mL).

**Fig. 4:**
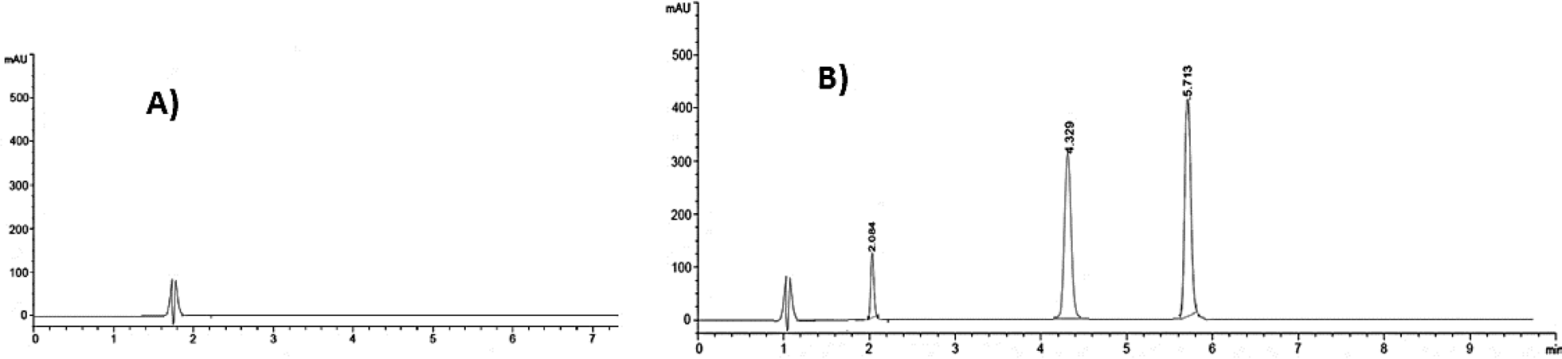
Chromatogram obtained by **A** blank urine and **B** spiked urine sample with AMX, ENR and metabolite (CIP)

We adopted the standard addition technique by adding (5.0, 10.0 and 20 µg/mL ENR) to initial known concentrations (5.0 and 45.0 µg/mL) to further validate the method, and we found that the results for determining ENR are closely aligned with its known content (Table 2) with no interference from AMX and CIP (Fig. 4).

### Statistical comparison with reported method

When comparing the results of ENR analysis obtained from our proposed HPLC method to those obtained from a reported method that is used to determine ENR [33], our computed t- and F-values were found to be below the critical values at a 95% confidence level. This demonstrates no significant differences in accuracy and precision between the two methods, as shown in Table 5.

**Table 5:** Statistical comparison between the results developed and the reported method* [33] for ENR determination

Parameter	RP-HPLC method-ENR	Reported method-ENR*
Mean	99.85	100.25
SD	1.220	1.290
Variance	1.488	1.664
n	9	6
Student’s t-test^a^	0.6088 (2.160)	
F-test^b^	1.118 (3.48)	

*RPHPLC using stationary phase (Kinetex® RP–C18, 150 × 4.6 mm, particle size 5 μm) and 0.002 M phosphoric acid/acetonitrile (83:17, v/v) as mobile phase

^a,b^The figures in the parenthesis are the corresponding theoretical values of t and F

### Greenness and whiteness evaluation

To quantify our greenness findings, we employed the GCC numerical tool and the AGREE graphical tool, and for whiteness assessment we used the RGB12 model. The comparison was done with one reported method of ENR determination in presence of AMX, using RPHPLC, stationary phase RP–C18 column with a gradient mobile phase of acetonitrile and phosphate buffer containing methanol at pH 5.0, a flow rate of 0.8 mL/min as mobile phase and dissolving the standards, lab mixtures and application in methanol [37].

**Fig. 5:**
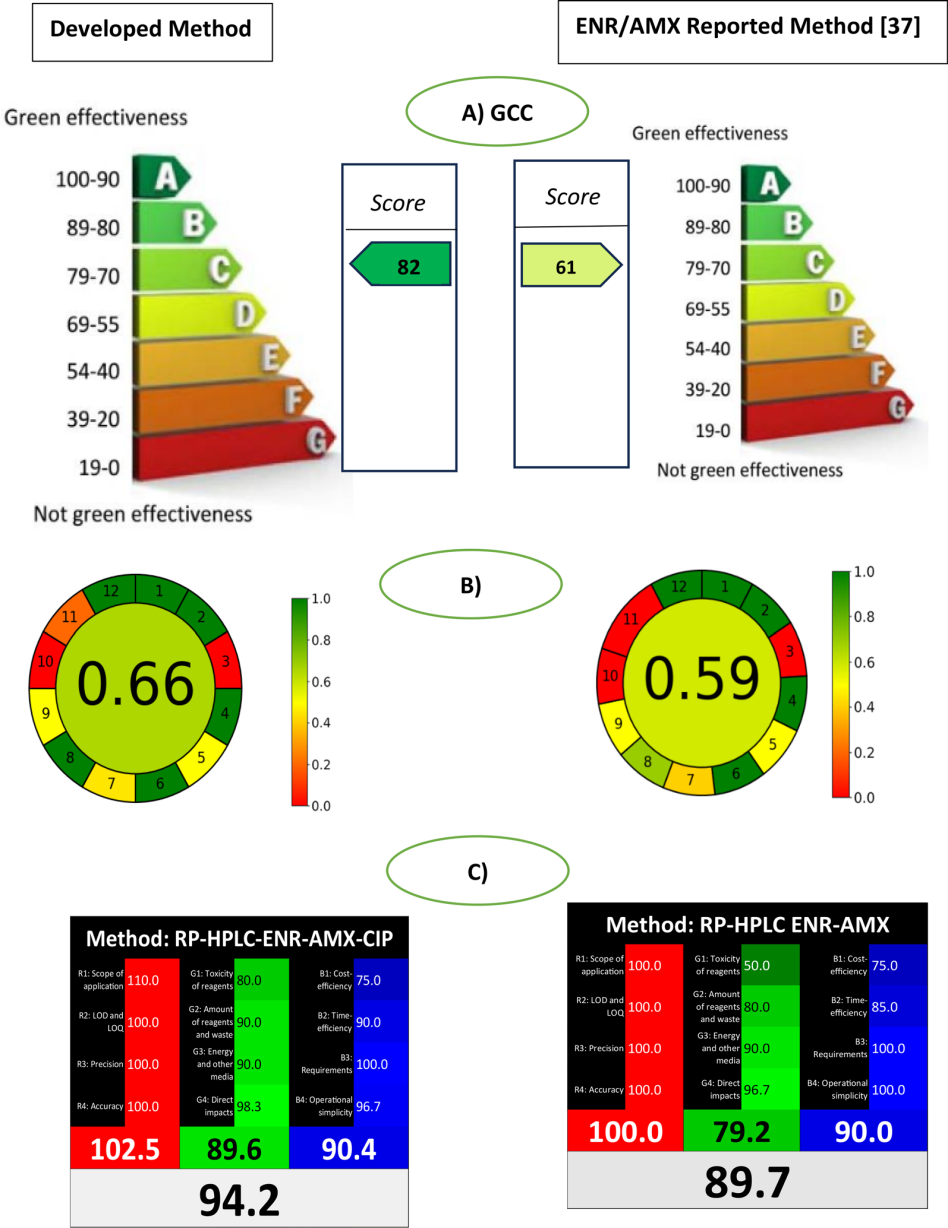
Greenness assessment comparison between the developed method and previous reported method using different green metrics. **A** GCC, **B** AGREE calculator and **C** RGB12 model

For the GCC metric, our developed method received a high score of 82 resulting in class B in green classification. Due to the little amount of the used solvent that is the greener alternatives to methanol and acetonitrile and the less amount of waste produced due to the faster time of analysis, and the collection of the wasted mobile phase to be reused and recycled. In addition, the wider application of our developed method compared to the reported one [37]. According to the GCC, the reported HPLC method is 61 light green score and in the green classification, it achieved class D. This results from the use of more noxious solvents like methanol and acetonitrile in the composition of the mobile phase, the quantity of the produced waste, as there was no recycling, and the analysis time was longer exceeding 10 min per run. The findings of the GCC were depicted in Fig. 5a.

By applying AGREE tool, it was found that the developed and the reported HPLC capture a little different greenness score. Our developed method scored (0.66) and the reported HPLC method scored (0.59). Figure 5b shows that both methods were labeled red because of their off-line nature and yellow due to the off-line testing samples, also noting their lack of miniaturization, and the amount of solvent used is more than 10 mL. The differences between the AGREE scores are not varying a lot, as both are chromatographic methods and the AGREE metric does not consider the characteristic of chemicals in terms of harm and volume, and waste generation and treatment like the GCC.

It is essential while developing analytical methods to ensure their effectiveness and accuracy in identifying low concentrations in samples. However, the criteria used by the GCC and AGREE metrics tend to overlook these essential aspects. To address this gap, we utilized the WAC tool. The results from the RGB12 model (Fig. 5c) demonstrate the precision and accuracy of our proposed method, which also boasts environmentally friendly features. When compared to previous HPLC reports, our approach showed wider scope of application, where determination in pharmaceuticals and biological samples was done in addition to the determination of the active metabolite. Due to the improvements in solvent sustainability and faster analysis time, the overall score for our developed method reached 94.2%. While the previous reported method used for comparison is considered less white for its lengthier process and greater solvent usage, resulting in lower ratings for its eco-friendliness and efficiency, it remains highly regarded for its precision, as demonstrated by a significant score in the red group. The combined whitening score for the reported HPLC reached 89.7%, as shown in Fig. 5c.

From an overall comparative view, our suggested method offers an eco-friendly alternative to determine ENR with wider application range; in pure form, pharmaceutical dosage forms and biological samples (urine) in the presence of a common co-administered medication (AMX) and its active metabolite with a very similar structure features (CIP) using only basic dilutions by a benign solvent mixture and simple treatment to eliminate matrix effect. Moreover, the developed method proved its applicability covering a lower concentration range for ENR determination (5.0–80.0 µg/mL) with the LOD 1.072 µg/mL, while the method used for comparison showed a range of (480.0–1120.0 µg/mL) with the LOD 0.074 mg/L, (Table 6). Besides, the system suitability testing showed higher resolution, selectivity, higher column efficiency for ENR with better peak symmetry.

**Table 6:** Comparison between the developed HPLC method and reported HPLC methods for analysis of ENR (with either AMX or CIP)

Method	Mobile phase	Analytes	Application	Run time	Linearity range	LOD/LOQ	Ref.
Developed method	Phosphate buffer pH 3.0: ethanol (90:10 v/v)	ENR, AMX, CIP	Pure powder, dosage form, urine	5.7 min	5.0–80 µg/mL ENR 5.0–100.0 µg/mL AMX 5.0–100.0 µg/mL CIP	LOD: 1.072 µg/mL ENR; 1.372 µg/mL AMX; 1.345 µg/mL CIP LOQ: 3.25 µg/mL ENR; 4.11 µg/mL AMX; 4.11 µg/mL CIP	
HPLC	2.0% aqueous formic acid–methanol–acetonitrile (75:13:12, v/v/v)	ENR, CIP	Bovine milk and plasma	8.15 min	1–100 ng/mL in plasma 2–100 ng/mL in milk ENR and CIP	LOD: NA LOQ: 2.0 ng/mL ENR, CIP in milk 2.0 ng/mL ENR, CIP in plasma	[32]
HPLC	0.002 M phosphoric acid/acetonitrile (83:17, v/v)	ENR, CIP	REPTILE PLASMA	12 min	2.0–100.0 μM ENR 3.0–100.0 µM CIP	LOD: NA LOQ: 2 μM ENR; 3 Μm cip	[33]
HPLC	75 mL methanol with 425 mL of 0.02 M KH_2_PO_4_, then adjusted to pH 5.0 with 2 M H_3_PO_4_	AMX, ENR	Injectable suspension	10 min	480–1120 g/mL AMX 240–560 g/mL ENR	LOD: 2.0 mg/L AMX; 0.074 mg/L ENR LOQ: 6.9 mg/L AMX; 0.24 mg/L ENR	[37]

## Concluding remarks

In summary, the rapid and precise RP-HPLC method we developed offers significant advantages, particularly in its ability to determine ENR in combination with AMX, as well as its active metabolite in various matrices, including pharmaceutical formulations and urine samples providing faster analysis time (less than 6 min). This advancement represents a major step forward in antibiotic monitoring, particularly in veterinary and environmental contexts. Our method was validated and proved to be accurate and specific with limit of detection of 1.072, 1.372 and 1.345 µg/mL for ENR, AMX and CIP, respectively.

Additionally, our method has been rigorously assessed using various green metric tools and demonstrated superior eco-friendliness compared to previously reported practices. It received a top classification under the Green Certificate Classification metrics and scored 82 (Level A) higher than the reported method that scored 61 (level D). Besides, its Analytical Greenness (AGREE) algorithm is 66, which is higher than the reported HPLC method. Moreover, our developed method scored 94.2 in the RGB12 model’s analysis of “whiteness” which is higher than the reported method (scored 89.7). By reducing solvent consumption and minimizing waste, our approach aligns with green and white chemistry principles, offering a sustainable solution for antibiotic residue analysis. However, while the method has many advantages, it also has some limitations. Being an HPLC method, it requires a large volume of solvents as we cannot fully get rid of it, yet we tried to minimize the use of hazardous ones, and the waste was collected to be reused and recycled. The method was validated according to ICH guidelines, proving its accuracy, precision, and specificity, making it an optimal solution for various applications in quality control, drug monitoring, and environmental laboratories.

## Data Availability

All data are available from the corresponding author upon request.
